# Letter by Namboodiri Regarding Article, "A Narrow QRS Complex Tachycardia With Apparently Concentric Retrograde Atrial Activation Sequence" - Alternative Mechanisms

**Published:** 2009-07-01

**Authors:** Narayanan Namboodiri

**Affiliations:** Department of Cardiology, Sree Chitra Tirunal Institute for Medical Sciences and Technology, Thiruvanathapuram, Kerala, India.

**Keywords:** Supraventricular tachycardia, accessory pathway, catheter ablation

Read with interest the article by Arias et al on a case of orthodromic atrioventricular reentry (ORT) mediated through the left free wall (LFW) accessory pathway(AP) with an apparently concentric atrial activation sequence [1]. Based on the observation that the atrial signals during the tachycardia were earlier in the His bundle (HB, anterosuperior septum) compared to the proximal coronary sinus (CS, posteroinferior septum), the authors proposed a de novo conduction delay at lateral mitral isthmus (LMI) in this case. However, the electrophysiological correlates of local conduction delay - fractionated electrograms or double potentials - were not documented across LMI to substantiate this. Though the retroaortic route potentially limited recording the endocardial atrial signals, no marked delay in the atrial electrograms between the adjacent CS bipoles was recorded even while CS was deeply cannulated. During ORT, prolonged local VA intervals in mid/proximal, but not in distal CS, bipoles could also represent a conduction delay in the posteroinferior left atrium (LA) immediately medial to the AP insertion. The given intracardiac tracings do not suggest this either. Furthermore, achieving a block across LMI is often difficult during ablative procedures due to the thickened local atrial tissue and /or blood flow of adjacent CS. In a large study, this necessitated epicaridal (inside CS) as well as endocardial ablation in 68%, and radiofrequency delivery exceeding 30 minutes in 20% of cases [2]. These characteristics do make a de novo conduction delay across LMI unlikely in an otherwise normal heart. Authors could have attempted atrial pacing at decremental cycle lengths through the distal CS bipole positioned lateral to LMI to unmask a potential functional delay across LMI and substantiate their hypothesis. This is expected to result in progressive displacement of the collision point of clockwise and anticlockwise impulses in the posteroinferior LA, finally leading to complete reversal of  the atrial activation sequence recorded in CS bipoles medial to LMI, in presence of the proposed functional conduction delay at LMI.

 However, this unusual electrophysiological finding merits discussion of possible alternative mechanisms. Though the authors could ablate the ventricular end of the AP at lateral mitral annulus, the atrial end was not located by the retroaortic mapping. No doubt, the atrial insertion site is the primary determinant of the atrial activation pattern during ORT. In a large series, 91% of APs did have an oblique transannular course, and the atrial end was found located leftward (lateral) to the ventricular insertion sites in nearly four-fifths of LFW pathways [3]. The earliest atrial activation in the second last bipole of CS with the decapolar catheter tip in the anterolateral mitral annulus in the index case locates the atrial end of the AP to the same quadrant, and is also consistent with this common insertion pattern. Furthermore, ORTs through the left anterior APs are known to have an earlier activation of HB compared to the proximal CS [4]. The substantial variability in the myocardial fibre orientation in the LA, as reported earlier on autopsy studies, and the preferential propagation of impulses closely correlated with the muscle fibre orientation could also contribute to the minor discrepancy in conduction time within the LA [5]. An even simpler explanation relates to the position of the catheter within CS. It is possible that the 7 Fr quadripolar catheter placed in CS, entered the ostium of a posterolateral cardiac vein. This could result in a misleading sequence of retrograde conduction as well as difficulty in advancing the CS catheter further within the main vessel as observed by the authors. Figure 1B lends some support to this notion by having a large ventricular electrogram in CS 1-2 compared to CS 3-4.
